# Forensic data management and database systems in forensic investigations for cases of missing and unidentified persons in Brazil

**DOI:** 10.1080/20961790.2022.2076994

**Published:** 2023-02-12

**Authors:** Melina Calmon

**Affiliations:** Grupo de Pesquisa em Antropologia Forense e Identificação de Pessoas, Academia Nacional de Polícia - Polícia Federal do Brasil, Brasilia, Brazil

**Keywords:** Forensic sciences, forensic data management, database, missing persons, unidentified persons, Brazil

## Abstract

Forensic investigations, especially those related to missing persons and unidentified remains, produce different types of data that must be managed and understood. The data collected and produced are extensive and originate from various sources: the police, non-governmental organizations (NGOs), medical examiner offices, specialised forensic teams, family members, and others. Some examples of information include, but are not limited to, the investigative background information, excavation data of burial sites, antemortem data on missing persons, and postmortem data on the remains of unidentified individuals. These complex data must be stored in a secured place, analysed, compared, shared, and then reported to the investigative actors and the public, especially the families of missing persons, who should be kept informed of the investigation. Therefore, a data management system with the capability of performing the tasks relevant to the goals of the investigation and the identification of an individual, while respecting the deceased and their families, is critical for standardising investigations. Data management is crucial to assure the quality of investigative processes, and it must be recognised as a holistic integrated system. The aim of this article is to discuss some of the most important components of an effective forensic data management system. The discussion is enriched by examples, challenges, and lessons learned from the erratic development and launching of databases for missing and unidentified persons in Brazil. The main objective of this article is to bring attention to the urgent need for an effective and integrated system in Brazil.

## Key points

Forensic investigations, especially those related to missing persons and unidentified remains, produce different types of data that must be managed and understood.

Brazil has had an erratic and poor performance in the search and identification of missing and unidentified persons. One of the reasons, and the main topic of this article, is the lack of a centralised data management system and database software that follow the steps necessary for them to be reliable and long-lasting.

There is a need for better institutional and national support, with the establishment of policies, financial resources, and full-time dedicated and trained staff.

The creation of a database for missing and unidentified persons in Brazil is crucial and will be challenging.

## Introduction

The term “missing person” is usually associated with the disappearance of someone under suspicious circumstances. However, in a wider sense, it must also include people whose whereabouts are unknown but whose safety is not a matter of concern [1].

Police agencies and other institutions each have their own criteria for classifying missing persons reports. The decision to establish how widely or narrowly to cast the missing persons net varies among agencies and differs among cities, states, and countries [1].

The complexity of a missing person investigation comes from the many categories in which law enforcement agencies, international institutions, and databases collect and release data on those cases. Haglund [2], notes that, unfortunately, for someone to be reported missing, they must first be missed, (and) many individuals who should have been reported missing are not missed and thus not reported. Hence, people whose disappearances go unreported by family or friends are unknown to investigative agencies. These aforementioned issues might obscure the real number of missing persons worldwide.

In Brazil, an accurate and realistic number of missing persons is unknown, as the country does not have databases for missing persons or unidentified remains. The number reported by the Brazilian Public Security Yearbook was 786 071 individuals between the years of 2007 and 2017 [3]. In the year 2020 alone, Brazil unofficially registered 62 857 cases of missing persons [4]; this number was reported by the Public Security Secretariats of the Sates, Civil Police, Military Police, Federal Police, and other sources of the Brazilian Public Security. Because Brazil lacks a centralised database with standardised data and national policies on the issue of missing persons, it is not possible to accurately determine how many cases have been solved or closed, nor how many individuals have been found alive or deceased. Moreover, although a definition for missing persons has been taken from the International Committee of the Red Cross protocols, Brazil does not have a classification of the types of missing persons. The types of missing persons are, for instance, the ones established by the National Crime Information Centre (NCIC; Table 1). In addition to this categorisation, the NCIC also requires that the demographics (e.g. age, sex, stature, and race) are collected and available before a case can be accepted. The lack of categories for types of missing persons limits research opportunities and the ability to establish who are the missing in Brazil. It further highlights the necessity for the development of policies and methodologies to solve this problem.

**Table 1.** National Crime Information Centre (NCIC) categories of missing persons.

Categories of missing persons	Description
Disability	A person of any age who is missing and under proven physical/mental disability or is senile, thereby subjecting him/herself or others to personal and immediate danger.
Endangered	A person of any age who is missing under circumstances indicating that his/her physical safety may be in danger.
Involuntary	A person of any age who is missing under circumstances indicating that the disappearance may not have been voluntary, for example, abduction or kidnapping.
Juvenile	A person who is missing and not declared emancipated as defined by the laws of his/her state of residence and does not meet any of the entry criteria set forth in the Disability, Endangered, Involuntary, or Catastrophe victim categories.
Catastrophe victim	A person of any age who is missing after a catastrophe.
Other	A person not meeting the criteria for entry in any other category who is missing and 1) for whom there is a reasonable concern for his/her safety or 2) who is under age 21 and declared emancipated by the laws of his/her state of residence.

The problem of deceased unidentified individuals is a global issue that has not received thorough attention. The marginalisation of this issue is caused by a variety of factors that vary by country. Ritter [5] described the missing persons situation in the USA as a “silent mass disaster” that is also a reality for Brazil. Worldwide, the most frequent and common problem is the lack of a consistent recording system of unidentified remains. This results in poorly organised records [6]. In forensic pathology and anthropology, the difficulties associated with cases of unidentified individuals are not well understood [7]. Historically, the problem with unidentified decedents is underestimated by the few publications that shed light on the issue [6, 8–12]. Lessa [13] suggested that modern society is characterised by the diminishing of family ties and the global migration of populations, which will only exacerbate this type of casework.

In Brazil, neither official nor unofficial data are available on the number of unidentified remains investigated by the Medico-Legal Institutes (Institutos Médico-Legais, IMLs). In 2007, a study determined that the remains of 7 287 individuals had been identified in five Brazilian states [13, 14]. Currently, it is estimated that 20 000 individuals are processed through the IMLs yearly. The remains of these individuals are either released or retained in custody as unidentified persons. The IMLs in Brazil do not share a standardised method of recording cases, including those concerning unidentified remains. In addition, the IMLs have been unable to provide for the storage of bodies, as many have limited room or facilities to keep the unidentified remains. COVID-19 aggravated the matter after funerary services, and the IMLs were overwhelmed with the bodies of deceased individuals in Brazil [15] because cemeteries did not have empty graves where the bodies could be buried. News agencies in Brazil have reported that the lack of spaces in cemeteries is a national problem [16–20]. Bodies of individuals who had been placed in mass graves due to the lack of cemetery space, are now being exhumed for proper analysis and data collection.

The main objective in the investigation of an unidentified individual, for ethical, civil, humanitarian, and criminalistics reasons, is to identify the decedent. Without this identification, many civil procedures cannot be completed (e.g. inheritance and insurance claims). If foul play has been established, the crime will be more difficult to solve.

Forensic scientists — anthropologists, pathologists, geneticists, odontologists, and others — are frequently confronted with cases of unidentified individuals. Medical examiner/coroner offices have reported incomplete or no data on unidentified remains cases [21]. Examples of incomplete data include an inconsistent collection of photographs, radiographs, identifying characteristics, fingerprints, DNA, as well as antemortem information about a case. The latter, a crucial part of any investigation, is usually derived from police reports and needs to be added to this multidisciplinary work, as identifications are made based on antemortem and postmortem data comparisons. When the work is deficient in any of these spheres, unidentified remains are buried in numbered “John/Jane Doe” graves, without a proper analysis and adequate data, and are likely to remain unidentified indefinitely [22].

Resources to aide in the identification of deceased unidentified individuals are becoming increasingly available worldwide. Some of these are multidisciplinary groups such as Operation Identification at Texas State University, whereas others are databases and protocols developed by international organisations such as the International Committee of the Red Cross. However, databases still face problems similar to those first diagnosed and exposed by Hofmeister et al. [23] including: institutional policy, legal framework and data protection, human resources and training, protocols and procedures, tools and software, and quality control and assurance.

Therefore, although evidence suggests that massive numbers of missing and unidentified persons would be positively impacted by the presence of an integrated database, it is important to consider the specific areas that need development and the implementation of such systems.

## Forensic data management

While considering the urgency of the creation of a database system that can manage information to facilitate the human identification process, it is important to consider all the requirements that are needed for such system to be effective. According to Hofmeister et al. [23], the following crucial components and their complexities should be considered.

### Institutional policy

The design, implementation, and long-term efficiency of a database relies on the creation and maintenance of institutional or national policies, their missions, and visions. Because it is important that a database is integrated for and accessible by a myriad of users, each institution must comprehend the utilities and limitations of the implemented system. In addition, the national or institutional policy must include a plan of action and strategy, with allocated human and financial resources.

### Legal framework and data protection

A national legal framework must be in place addressing the rights of missing persons, deceased individuals, and their families. Whenever human rights violations are investigated, the country must have mechanisms that allow for the prosecution of these crimes; in contrast, strictly humanitarian identifications^1^ must also be allowed and protected under the legal framework.

The data collected on missing and unidentified persons investigations are sensitive and confidential, as they may include personal information and biological samples of family members. Therefore, the protection of personal data, human remains, and genetic information, must be put in place. These data protection standards must be followed throughout the different layers of the database system.

### Human resources and training

Human resources must be allocated for the adequate management of data and the database system. Such resources must be framed in the institutional or national policy. Hiring and training of personnel are crucial for ensuring long-term efficiency of the database system. In addition, it is important to ensure that human resources comprise data collectors, data entry staff, data analysts, and information technology support, to name a few.

Staff in charge of collecting information (both antemortem and postmortem) need to understand the reasons for its collection, and comprehend the use, interaction, and impact of the data they collect. Collectors of antemortem data also need to be trained in psychosocial skills, as they will be interacting with families and need to promote trust and guarantee a safe environment in which family members can provide information. Collectors of postmortem information must be experts in their field and follow best practices and recognised scientific methodologies. The staff responsible for data entry need to be aware of specific technical and scientific terminologies, promoting and ensuring consistency throughout their data input. The data analysis personnel need to not only understand the data, but also the tools available within the data management system, guaranteeing that the data are serving their purpose and achieving the expected results. In addition, this specialised staff is responsible for task such as producing technical reports, statistical analysis, and interpretation of the data for contextual understanding. Information technology support professionals are responsible for gatekeeping the security of the data, data backup, and the distribution and access of data amongst the different users, agencies, and institutions.

Continuing education and training must be regularly provided to the personnel responsible for data collection and data entry to ensure standardisation and quality control.

### Protocols and procedures

The standardisation of processes, methodologies, and procedures is crucial for the entire forensic system to work properly. Standard operating procedures (SOPs) expose and clarify the roles and responsibilities of the data management system, staff, and organisations and institutions involved in its usage. Broadly distributed interagency and interdepartmental SOPs promote high levels of cooperation amongst the parties involved.

Moreover, information must be centralised to ensure quality control, work processes, and data flow. The specific departments that build the data management system must be included in the developed protocols and procedures; for example, the protocols on psychosocial support for families, antemortem data collection, postmortem data collection, data entry, use of the data software, and data analysis.

### Tools and software

The database software, combined with the previously mentioned components, can promote standardisation and assist with the work of large caseloads by providing a computerised system. Highly specialised software such as geo-referencing and mapping tools, DNA software, fingerprint matching software, and custom software such as programmes for missing persons registries and evidence management need to be integrated.

Because a system needs to be integrated, it is imperative that the data can be exchanged to avoid duplications or inconsistencies and to provide for the compilation of information on a specific case. A database should be thought of as one of the tools needed for facilitating an identification and not as a tool that will generate the identification. An identification should only be carried out by experts who compile information and produce scientifically based reports that support an identification. Identifications should only be made in cases where the antemortem and postmortem data are consistent in sufficient detail to conclude that they are from the same individual to the exclusion of all others.

### Quality control and assurance

For a database software and its associated tools to be reliable and verifiable, a high standard of quality control and assurance must be met. This is important so the quality and standards are maintained throughout use of the system.

A quality control process should include a multilevel implementation including a self-review and an anonymous beta-testing phase. This process increases the likelihood that inconsistencies and errors are caught prior to the database going live. Database maintenance and regular reviews of data must be scheduled for data cleaning, which is essential to prevent duplications, ensure consistency, and identify any bugs or technical issues.

The analysis, storage, and reporting of the data must also undergo regular reviews to ensure that goals are being achieved.

## Search for the missing and unidentified persons in Brazil

The IMLs in Brazil are public organisations under the State Secretary of Public Security (Secretaria de Estado de Segurança Pública) and are considered to be an arm of law enforcement. Each state of Brazil has their own IML, with other departmental branches to cover the different regions in each state.

The IML produces the medico-legal reports on live individuals, cadavers, human body parts, and complete or incomplete skeletonised remains. The institute also provides complementary laboratory exams in pathology, toxicology, forensic chemistry, and forensics on sex crimes when required by the judiciary system or law enforcement agencies. These laboratory reports are usually necessary for police reports as well as for judiciary and administrative investigations [24]. As a technical-scientific service provider at the disposal of the police and judiciary, the IML issues confidential reports that become a fundamental piece of investigations, police inquiries, and other legal matters [24]. IMLs usually do not maintain webpages or databases for missing or unidentified persons except for a few specific institutes such as the ones in the state of Pará and in the city of Belo Horizonte. However, these resources are not available to the general public. The IMLs of Curitiba, in the south of Brazil, and the city of Sao Paulo provide an online list of their identified but unclaimed bodies in an effort to release information about individuals to their families [24].

In Brazil, law enforcement agencies at the state and local levels that carry out detective work, forensics, and criminal investigation are called the Civil Police. The Civil Police is an organisation under the Secretary of State Justice and the Secretary of Public Safety. Each state of Brazil has its own Civil Police force, which yields a total of 27 agencies: one for each of the 26 states and one for the Federal District. The services of the Civil Police are available through police stations, which are agencies that have jurisdiction in small cities or neighbourhoods of the big cities. The state Civil Police forces have their individual websites where they provide access to information from their Missing Persons Police Agencies (Delegacias de Polícia de Pessoas Desaparecidas, DPPD) or Homicide and Protection of Persons Police Agencies (*Delegacias de Polícia de Homicídios e de Proteção à Pessoa, DPHPP*). These specific agencies deal with the report and investigation of missing persons cases, and many allow the public to register a case *via* their online police agency website [1].

One of the main issues with the DPPDs and DPHPPs is that many of their systems are not interconnected, even when different departments are in the same state. Moreover, an agency in one state does not, as a rule, receive or send information to another agency in another state. The jurisdiction of a missing persons investigation is usually limited to the place where the missing person’s family or friends reside.

A brief search for the topic “missing persons’ NGO” (ONG pessoas desaparecidas in Portuguese) reveal many links containing information about NGOs that can provide support in the search for missing persons in Brazil. When accessing the links, however, the websites are outdated, or the projects were discontinued because of a lack of funding or personnel. Many websites are unavailable.

Brazil has been erratic and slow in the search and identification of missing and unidentified persons. One of the main reasons is the lack of a centralised data management system and database software. Governments have tried and failed numerous times to develop and implement a robust system that can interconnect the different agencies involved in this type of investigation.

### Location and Identification Programme for the Disappeared

In 2012, the Rio de Janeiro State Public Ministry (Ministério Público Estadual, MPRJ) established the Location and Identification Programme for the Disappeared (Programa de Localização e Identificação de Desaparecidos, PLID). The programme was originally established in 2012 under the National Public Ministry Council (Conselho National do Ministério Público, CNMP) and was known as the Victim Identification Programme (Programa de Identificação de Vítimas, PIV). The objective of the PIV was to investigate homicide cases in which victims could not be identified and who were classified as missing persons. However, because many disappearances were found to be non-criminal, and because of the broader spectrum of jurisdiction maintained by the Public Ministry, the MPRJ transformed the PIV into the PLID in 2012.

The PLID is a repository of data originating from different agencies. The programme’s goal is to aid in the resolution of disappearances occurring in the state of Rio de Janeiro that involves disappearances of both a criminal and non-criminal nature.

Therefore, the PLID has three types of registries: missing persons, institutionalised persons, and unidentified human remains. Each registry has its own distinctive process of entry, but the ultimate goal is to combine existing information on missing persons cases that would otherwise be held independently by different agencies and institutions. To achieve that goal, the PLID integrates databases and public and private documents to match specific information that can lead to the identification of missing individuals. Generally, the missing persons reports originate from police agencies, hospitals, clinics, and treatment institutions (such as hospices and mental health facilities). Unidentified human remains reports usually originate from police agencies and information sent to the PLID from organisations such as the IMLs.

In August 2017, the PLID became nationalised after it merged with CNMP and became known as the National System for the Location and Identification of the Disappeared (Sistema Nacional de Localização e Identificação de Desaparecidos, SINALID). The mission of this programme is to maximise the integration and availability of data to facilitate the resolution of missing persons and human trafficking cases. It also aims to implement protocols for managing data from a national perspective. The programme established technical cooperation and partnerships between agencies to provide systematic inter-organisational communication. Additionally, SINALID is responsible for providing statistics and analysis that can be used to shape public policies.

In February 2018, the CNMP and the Supreme Electoral Court (Tribunal Superior Eleitoral) partnered to provide the biometric information of more than 80 million Brazilians to SINALID. In August 2018, the CNMP and the Public Security Ministry (Ministério de Segurança Pública) signed a cooperative agreement to expand SINALID. This agreement will allow the sharing of data from the Unified System of Public Security (Sistema Único de Segurança Pública) and SINALID [25, 26]. Until June of 2020, SINALID had assisted in 10 082 cases of missing persons^2^.

### Caminho de Volta

The project Caminho de Volta was developed by the School of Medicine at the University of Sao Paulo (FMUSP) in 2004 as a partnership with the PLID in Sao Paulo [27]. The project offered the free collection and storage of DNA and biological material for the families of missing persons under 18 years of age. The DNA and biological material were maintained in a database for the purpose of matching the missing persons’ DNA with family reference samples [28]. The programme had jurisdiction in the state of Sao Paulo and was recognised by the Sao Paulo State Public Ministry for use by the Justice State Court in cases of unidentified adolescents or children.

In addition to its collection and storage services, Caminho de Volta offered psychological and social services to the families of the missing. Moreover, the project used insight gained from interaction with family members to understand the factors related to missing persons cases to advocate for public policies to prevent or minimise the number of children and adolescent disappearances [28].

At this time, the website of the project has not been updated since 2017, and most of their reported services stopped in 2014. The FMUSP group responsible for the project had no desire to renew the partnership with the state of Sao Paulo, according to a personal communication with the former State Secretary of Public Safety of Sao Paulo. However, steps are being taken to re-establish such a collaboration, aiming for a possible integration with SINALID.

### Integrated Network of Genetic Profiles Database (RIBPG)

In Brazil, the forensic use of DNA began in 1992 with the Civil Police of the Federal District (Polícia Civil do Distrito Federal, PCDF), which initiated its own laboratory for DNA research. However, it was not until 1994 that the first case involving forensic DNA came before the Brazilian justice court. In that year, two members of the PCDF were sent to the USA to analyse the DNA related to the investigation of two crimes that occurred in Brasilia [29].

The limited use of forensic DNA analysis in Brazil led the National Secretariat of Public Security (Secretaria Nacional de Segurança Pública, SENASP) to develop and increase the use of DNA analysis. SENASP initiated standardising procedures, provided new laboratory equipment, trained personnel, and provided their documents, projects, and norms available in a digital form. Two of the most important documents available through this programme were the *Standardization of DNA Exams in Criminal Settings* (*Padronização de exames de DNA em perícias criminais*) and *Resolution SSP number 194/99*, which established the norms and procedures for the collection and analysis of biological material for human identification [30].

The collection of biological material in Brazil started with the use of CODIS, which was made available to federal and states laboratories through an FBI donation. In 2010, CODIS was installed in 18 state laboratories in Brazil and one federal laboratory [31]. In 2012, Law no. 12.654 authorised the collection and use of biological material for the purpose of criminal investigation and the creation of a DNA database [32, 33]. The creation of the Integrated Network of Genetic Profiles Database (Rede Integrada de Bancos de Perfis Genéticos, RIBPG) in Brazil is a partnership between the Ministry of Justice and the Public Safety Secretariats of the States in an effort to provide the genetic profiles of judicial interest to all official investigation laboratories. RIBPG was destined to be used for the criminal collection and identification of missing persons [34–36].

The existence of RIBPG in Brazil allowed for the creation of a national campaign for the collection of genetic material from the families of missing persons, which occurred in June of 2021. The campaign happened in an integrated manner in every state of Brazil, with the collaboration of the Civil and Federal Police [37]. It was the first time that a national collaborative effort was developed focusing on the missing persons issue in Brazil. The RIBPG is one of the largest networks of criminal investigation laboratories in the world, putting Brazil in the group of more than 60 countries who use DNA databases for investigation.

### Desaparecidos.gov

The website desaparecidos.gov was created in 2010 and contains information about missing Brazilian children and adolescents. In 2009, Law no. 12.127/2009^3^ created the National Register for Missing Children and Adolescents. In 2010, the Human Rights Secretary of the President of the Republic (Secretaria de Direitos Humanos da Presidência da República), in partnership with the Justice Ministry, developed the National Database for Missing Children and Adolescents. This database was designed to help in the investigation and location of missing children and adolescents in Brazil. The database allowed the registration, search, and dissemination of information about cases and involved many federal policy maker institutions. Any public, private, for-profit, or not-for-profit organisation could register a case of a missing child or adolescent.

Since its creation, the database has received 957 registrations for missing children and adolescents. Unfortunately, the website and the database have been deactivated from its online host for more than 2 years now. The Human Rights Ministry, the institution responsible for the publication of data, reports that the website is being updated. The upgrades are due to the establishment of a partnership between the National Register and the International Centre for Missing and Exploited Children, which will add Brazil to the Global Missing Children’s Network. According to the Human Rights Ministry, the database improvements will include a better family service system, better information dissemination, and improved management of case input, along with integration with Federal and Civil Police agencies. No firm date has been provided for the completion of the upgrades.

### National Registry of Missing Persons

The proposed Bill no. 6.699/2009^4^ was intended to create the National Registry of Missing Persons, which would contain the physical characteristics and photos of missing persons, together with the contact information for the family or agency responsible for including the missing persons case in the database. The bill also required federal institutions and agencies to cooperate and establish a way to access the information in the database as well as a process for validating and updating the data. Finally, it demanded that the costs related to the development, installation, and maintenance of the database come from the resources available in the National Public Security Fund.

This original bill was transformed into Ordinary Law no. 13.812/2019^5^, which instituted the National Policy for Searching of Missing Persons and created the National Registry of Missing Persons. The law states that the search for and location of missing persons is considered an urgent priority by the government and should preferably be carried out by specialised investigative bodies, with mandatory operational cooperation through a national registry for including public security bodies and other entities that may intervene in these cases.

In relation to the database, Article 5 of the law establishes that the National Registry of Missing Persons, which aims to implement and support the policy referred to in this Law, will be composed of:

- *A public information database, freely accessible through the Internet, with information about the physical characteristics of missing persons, photos, and other information useful for their identification whenever there is no risk to the missing person’s life;*
- *A confidential information database, intended for public security agencies, with standardised records of each occurrence and the number of the police report, which must be the same as the police investigation, as well as information about the physical characteristics of the missing persons, photos, contacts of family members or persons responsible for including the missing person’s data in the registry, and any other information relevant for their prompt location;*
- *A confidential information database, intended for public security agencies, which will contain genetic and nongenetic information on missing persons and their families, exclusively intended to find and identify the missing person.*

The discussions for the development of the protocols, procedures, and systems have already started. Working groups composed of specialised professionals are creating guidelines to be presented and discussed nationally. The future system is expected to allow for the sharing and access of information in different levels among actors; however, the integration with already existing systems has not been mentioned at this development stage.

## Discussion

The Brazilian national framework assigns the investigation of missing persons cases to national and state authorities. Yet in practice, there are impediments that delay and sometimes prevent these investigations from taking place. Successful identification requires a systematic collection and management of different types and scales of data. An identification will then typically occur when postmortem data from a body and antemortem data from the families of the missing person are brought together for comparison.

For a forensic database system to be effective, there must be coordination and supervision of all the actors involved while maintaining a national database on missing and unidentified persons and liaising with the families of missing persons. These features promote the sharing of information with other actors and institutions involved in these investigations.

Currently there is a clear conflict between expectations, legal duties, and reality. While addressing the requirements for the success of a forensic database system, we recognised that we cannot expect institutions and agencies that are under-resourced (with respect to both money and human resources) to stretch their staff into a long-term and time-consuming process while also continuing their daily investigative work. Therefore, there is a need for better institutional and national support, with the establishment of policies, financial resources, and full-time dedicated and trained staff.

While the current response in Brazil is largely unable to address the issues of missing and unidentified persons, there are international examples of good practices being implemented, especially regarding the development and use of forensic, data management, and database systems. Different nations have shown that it is possible to investigate missing persons cases and identify unidentified individuals when given appropriate resources and commitment by their institutions, states, and country.

A collective summary of the main shortcomings of the existing database management systems is as follows:

1. Problems with case data collection, chain-of-custody, evidence tracking, and overall fragmentation of the collected information;
2. A multiplicity of databases (governmental and non-governmental) that do not send data to a centralised system;
3. Barriers and difficulties for the participation of family members during the investigation (e.g. antemortem data collection, DNA reference samples, and overall knowledge about efforts on the case);
4. A general lack of or deficient antemortem data;
5. Lack of a mechanism for exchanging information on missing and unidentified persons;
6. IMLs that do not exchange information as a routine practice;
7. IMLs that do not share a standardised protocol for practices and exams (e.g. autopsies and anthropology, odontology, or radiology exams);
8. DNA comparisons are only done on a case-by-case basis, as opposed to a large-scale effort;
9. Lack of clear statistics on the number of missing persons and unidentified remains;
10. Disparity in the availability of specialised forensic services and accessibility by families of missing persons;
11. Lack of a standardisation of methodologies and forensic procedures for the identification of human remains by forensic practitioners;
12. Disparities in the processes for postmortem data collection prior to the burial of unidentified remains, and deficiency in the proper burial and registration of unidentified remains in cemeteries.

In addition to these problems, other issues that affect the identification process of unidentified individuals have been observed:

- The individual was not reported missing or it is not known to be missing;
- The remains were found in another jurisdiction, and the inability of databases to connect with each other prevents a match between missing and unidentified persons cases;
- Biological material and auxiliary exams were not performed to collect information on the unidentified case;
- The biological profile methodologies miscategorised the individual in one or more elements and a match is improbable unless a correction is made;
- The missing persons report was filled incorrectly;
- The law enforcement agency was backlogged with cases with higher priorities.

## Conclusion

Data management systems are becoming an indispensable part of forensic investigations, particularly in large-scale investigations such as those regarding missing and unidentified persons. A set of good practices, whenever implemented, will result in quality data and enable the comparisons needed for a forensic identification.

It is important to keep two main factors in mind when discussing and implementing a forensic data management system. First, a data management system is a multifaceted concept and not a limited software tool. It starts from the collection of data, assurance of data quality, definition of workflows, sharing of data, protection of data, and the centralisation and appropriate analysis of the information inserted and transferred to the system. Second, an effective forensic data management system needs to be supported by the implementation of policies, training, quality support and control, and financial support. This framework, or mechanism, requires time and resources, a focus on the standardisation, access, and distribution of data as well as guarantees that the data can be tracked, checked, and that a chain of custody for the data is maintained. When a forensic data management system is implemented correctly, the forensic and investigative processes are improved and the network of actors who work on such cases are better equipped to communicate and collaborate on investigations. The families of missing and unidentified persons are also allowed to more actively participate in the investigations, are better aware of the resources, and are better informed of the current status of the investigation.

The creation of a database for missing and unidentified persons in Brazil is crucial. However, there will be challenges. For the database to be efficient, it must allow access — at different levels — to investigators and the public. Brazil is inhabited by many native communities located far from city centres. To reach every Brazilian community, the collection of cases needs to be proactive, with investigators contacting rural and isolated communities. Additionally, with the advances in forensic technologies, law enforcement agencies have become increasingly interested in revisiting cold case investigations. Cold cases must also be started proactively by medico-legal investigators.

For every body without a name, and for every name without a body, there are families and friends whose lives are deeply affected by the ambiguous loss of their loved ones.

## Notes

1 “Humanitarian identification” is here used to refer to cases where the only goal of the investigation is the identification of the deceased individuals; no prosecution of crimes is sought.

2 According to the website https://www.cnmp.mp.br/portal/institucional/comissoes/comissao-de-defesa-dos-direitos-fundamentais/sinalid/informacoes-sobre-o-sistema#:∼:text=Mais%20do%20que%20um%20sistema,nacional%20de%20descoberta%20de%20paradeiros.

3 Law number 12.127/2019: http://www.planalto.gov.br/ccivil_03/_ato2007-2010/2009/lei/L12127.htm

4 Proposal of Bill number 6.699/2009: https://www.camara.leg.br/proposicoesWeb/prop_mostrarintegra;jsessionid=653570A44285713BC7033F5478057 51A.proposicoesWeb1?codteor=731665&filename=Avulso+-PL+6699/2009

5 Law number 13.812/2019: http://www.planalto.gov.br/ccivil_03/_ato2019-2022/2019/lei/L13812.htm
